# Recent Updates in the Treatment of Erythema Multiforme

**DOI:** 10.3390/medicina57090921

**Published:** 2021-09-01

**Authors:** Alexa Soares, Olayemi Sokumbi

**Affiliations:** 1 Mayo Clinic Alix School of Medicine, Mayo Clinic, 200 First Street SW, Rochester, MN 55905, USA; Soares.Alexa@mayo.edu; 2 Department of Dermatology, Mayo Clinic, 4500 San Pablo Rd S, Jacksonville, FL 32224, USA

**Keywords:** Erythema multiforme, treatment, updates

## Abstract

Erythema multiforme (EM) is an immune-mediated condition that classically presents with discrete targetoid lesions and can involve both mucosal and cutaneous sites. While EM is typically preceded by viral infections, most notably herpes simplex virus (HSV), and certain medications, a large portion of cases are due to an unidentifiable cause. EM can be confused with other more serious conditions like Stevens–Johnson syndrome (SJS); however, clinical research has provided significant evidence to classify EM and SJS as separate disorders. Treatment of EM is highly variable, depending on the etiology, the involvement of mucosal sites, and the chronicity (acute vs. recurring) of the disease. If the etiology or causal medication/infection is identified, then the medication is stopped and/or the infection is treated prior to initiating symptomatic treatment. Treatment for acute EM is focused on relieving symptoms with topical steroids or antihistamines. Treatment for recurrent EM is most successful when tailored to individual patients. First line treatment for recurrent EM includes both systemic and topical therapies. Systemic therapies include corticosteroid therapy and antiviral prophylaxis. Topical therapies include high-potency corticosteroids, and antiseptic or anesthetic solutions for mucosal involvement. Second-line therapies for patients who do not respond to antiviral medications include immunosuppressive agents, antibiotics, anthelmintics, and antimalarials

## 1. Introduction

Erythema multiforme (EM) is a cutaneous, and sometimes mucocutaneous condition that is typically precipitated by viral infections, most commonly herpes simplex virus (HSV), and the use of specific medications [1,2,3,4]. In many cases, however, the inciting factor for the development of EM remains unknown. It classically presents as numerous targetoid lesions with concentric rings of distinct color variation in an acral distribution [1,2,3,4,5]. Target lesion appearance can differ from patient to patient, and frequently, both typical (lesions with three concentric rings) and atypical (lesions with only two concentric rings) lesions are seen. Prodromal symptoms usually do not accompany EM; however, in cases where there is mucocutaneous involvement, prodromal symptoms have been observed [1,2,3,4,5,6]. Lesions usually erupt over a 72-hour period, and in some cases, produce a mild pruritus or “burning” sensation [1,2,3,4].

There can be occasional mucosal involvement in EM, which is what splits the condition into its two broad categories: EM minor (a form with no mucosal involvement) and EM major (a form that includes mucosal membrane involvement) [1,6,7]. The most common mucous membranes that are involved include the lips, tongue, and the buccal oral mucosa. Genital or ocular mucosal lesions have also been observed, as well as any combination of the mucosal sites listed [1,6,7]. Historically, EM major was thought to be part of a class of conditions that included Stevens–Johnson syndrome (SJS) and toxic epidermal necrolysis (TEN). However, clinical literature over the past decade has provided significant evidence that supports EM major as a completely separate condition from SJS that share similar mucosal lesions but distinctly different cutaneous lesions [6,7]. Another distinct entity from EM is Rowell syndrome (RS), a clinical triad of EM-like lesions, lupus erythematosus, and particular immunologic patterns such as speckled pattern of antinuclear antibody (ANA), positive anti-Ro/SSA or anti-La/SSB, and positive rheumatoid factor (RF), all of which are required to meet the diagnostic criteria for RS. It is important to note that EM as a condition is not associated with any specific immunologic pattern or serologic abnormalities typically found in autoimmune diseases [8].

While acute disease is usually self-limiting, some patients experience recurrent disease. Identifying the etiology of EM is crucial in developing a successful treatment modality [1,2]. Most acute cases of EM have been reported to stem from infections caused by HSV and *Mycoplasma pneumoniae*. A more recent association between EM and severe acute respiratory syndrome coronavirus 2 (SARS-CoV-2) pathogen, the novel coronavirus responsible for the recent pandemic, has been described [9,10]. Medications that have been implicated in causing EM include nonsteroidal anti-inflammatory drugs (NSAIDs), sulfonamides, antiepileptics, and antibiotics [1,2,3]. Recurrent cases of EM have also been likened to HSV and *Mycoplasma pneumoniae* infections, as well as to hepatitis C infections, and vulvovaginal candidiasis [1,2]. Other associations include menstruation, complex apthosis, and a high dietary intake of benzoic acid (a food preservative) [1,4,11]. It is unclear how many cases of EM that are initially determined to be idiopathic actually have an underlying or subclinical infection with HSV [12,13,14].

A Mayo Clinic series of 48 patients diagnosed with recurrent EM suggested that HSV infection was the most common cause; however, over 60% of patients were determined to have idiopathic recurrent EM [1,3]. A few studies have put forth the possibility that a subclinical infection is likely in many cases of idiopathic recurrent EM [1,12,13,14]. For example, polymerase chain reaction (PCR) has identified HSV DNA in the skin biopsies of 3 out of 5 patients with idiopathic EM [13]. An additional study identified HSV DNA was present in the biopsies of 6 out of 12 patients with idiopathic EM [14].

A rarer type of EM is known as persistent EM, which is defined by the continuous appearance of EM lesions with marked resistance to therapy. Lesions are typically widespread and are, by definition, uninterrupted [1,15,16]. Cases of persistent EM have been associated with underlying malignancies, inflammatory bowel disease, as well as infections with Epstein–Barr virus, cytomegalovirus, hepatitis C virus, and influenza [15,16]. A summary of well-documented trigger factors is outlined in Table 1.

**Figure 1:**
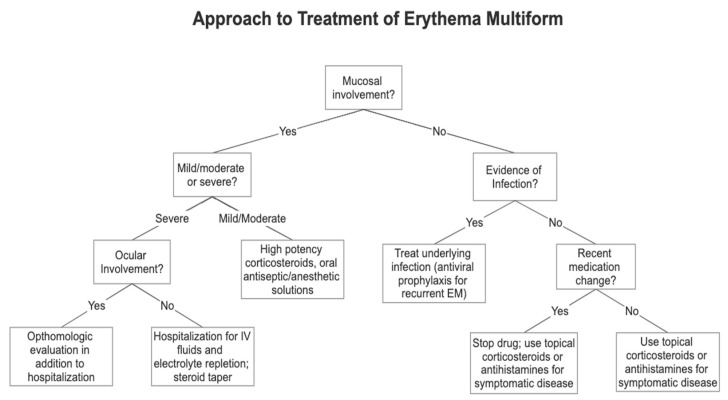
This flowchart details the clinical approach to treating each type of EM based on the clinical features of mucosal involvement, severity of disease, and infection or drug association. Adapted from “Erythema Multiforme: Recognition and Management” by Trayes et al. [2] with permission from the American Academy of Family Physicians, © 2021.

Treatment modalities differ for acute and recurrent disease. In acute disease, treatment is rarely needed as the lesions will typically regress over the course of several weeks, and supportive treatment is focused on improving symptoms [1,2,3]. In recurrent EM, treatment focuses on addressing the etiology through systemic antiviral prophylactic therapy. Refractory or resistant disease is more difficult to treat, generally relying on systemic immunosuppression [1]. A schematic on how to approach the clinical treatment of EM is outlined in Figure 1. This review will provide an overview on the treatment of EM, focusing on the newest evidence (limited to clinical studies published after 2001).

## 2. Updates in Treatment

Most of the treatment recommendations for EM are based on small case series or expert opinion. There have been few clinical trials [17]. Prior to treatment, the etiology should be determined. If there is evidence of a recent infection, then treating the infection is the first step in management. Similarly, if there is evidence that the EM is caused by a medication, discontinuing the medication is the initial step [1,2,17]. Once the etiology has been addressed, acute EM can be managed with topical steroids or antihistamines, if needed to improve symptoms. In the case of HSV-induced EM, some experts recommend early intervention with oral acyclovir to reduce disease duration and symptomaticity [1,2,3,17]. However, current evidence is limited in supporting the hypothesis that early antiviral therapy reduces the time to symptom and lesion resolution [17,18]. Table 2 provides an overview of the first-line therapy for each type of EM and the special considerations that need to be evaluated in every case.

## 3. Acute EM

Treatment for acute or isolated cases of EM typically do not need intervention, but in cases where patients are experiencing uncomfortable symptoms, topical steroids, antiseptics, and oral antihistamines are recommended. In acute HSV-induced EM, antiviral suppressive therapy can be used, however, several studies have suggested that the administration of antiviral therapy in this context does not alter the clinical course of the disease [4,5]. In EM preceded by an *M. pneumoniae* infection, antibiotics can be considered, but again, the goal of treatment should be symptomatic relief [1,2,3,4,17,18].

EM has been described following SARS-CoV-2 infection. However, in most of the cases reported, the medications used to treat the underlying infection could not be excluded as potential causes for the EM-like lesions [9,10]. Treatment in these cases consisted of stopping viral drug therapy and starting a tapered course of methylprednisolone [19].

## 4. Mucosal EM

Treatment for EM with mucosal involvement largely depends on the degree of severity. In mild or moderate disease, high-potency topical corticosteroid gel is used along with oral antiseptic washes and oral anesthetic solutions [1,2]. In severe disease with extensive mucosal involvement, hospitalization is generally recommended due to limited oral intake. Administration of intravenous fluids and electrolyte replacement are recommended. Additionally, systemic glucocorticoid therapy may be used, most commonly, prednisone 40–60 mg/d, tapered over 2–4 weeks [1]. If ocular involvement is suspected, then ophthalmologic consultation is necessary to prevent serious future complications. Ophthalmologists may prescribe ophthalmic medications, such as antibiotic eyedrops, corticosteroid eyedrops, and topical ophthalmic lubricants to aid in recovery and symptom resolution [20].

## 5. Recurrent EM

Recurrent EM is the most difficult type of EM to treat due to its refractory nature. In both HSV-associated EM and idiopathic EM, the first-line treatment is antiviral prophylaxis [21]. Current recommendations include acyclovir, 400 mg, twice daily, valacyclovir, 500 mg, twice daily, or famciclovir, 250 mg, twice daily [1]. These medications can be administered orally in either a continuous or intermittent fashion [12,18]. A randomized controlled trial from 1995 outlined that the most effective approach to treatment was continuous oral antiviral therapy for a period greater than six months [18]. The greatest efficacy of antiviral therapy is observed in patients whose disease has a clear association with HSV infection. The goal of treatment is to reduce the number of recurrences and to induce remission, which is difficult to maintain. Recurrence is frequent once antiviral therapy is stopped [1,2].

One study showed that out of 15 patients diagnosed with HSV-associated recurrent EM, only 4 remained in remission after the 6-month continuous antiviral therapy was discontinued [18]. Patients who are responsive to antiviral therapy should be treated for a minimum of 1 to 2 years before the therapy is discontinued. If there is recurrence after therapy discontinuation, the medication should be initiated again at the lowest effective dose. Discontinuation can be trialed again after 6–12 months of restarting therapy [1].

Patients with recurrent EM that are unresponsive to antiviral therapy can try other antiviral drugs or double the dosage of the current drug. Additionally, other systemic agents may be used [1]. These treatments are outlined in Table 3.

Antibiotics, azithromycin, and dapsone specifically, have both produced clinical improvement in patients with recurrent EM. Complete response to dapsone was observed in 6 out of 13 patients in a case series from 2017 [22]. However, 3 patents in this series had to stop treatment with dapsone due to side effects. Another series from 2010 showed evidence of a complete response to dapsone in 3 out of 9 patients [3].

A small case series reported complete response to thalidomide in 6 out of 7 enrolled patients who had been diagnosed with persistent EM major and were resistant to treatment with acyclovir and corticosteroids [23]. More recently, a case report from 2008 included a single patient who had an excellent clinical response to thalidomide after 6 months of persistent EM that was unresponsive to valacyclovir [24].

An observational study from 2018 demonstrated that levamisole showed significant reduction in EM recurrence in 23 patients, compared to standard therapy with corticosteroids and antiviral therapy [25]. Other immunosuppressants, including mycophenolate mofetil, have been less successful, with a complete response in 3 out of 8 patients as reported by a systematic review of the treatment of EM in 2019 [17].

Additionally, a single patient with recurrent EM had a rapid and complete response to treatment with adalimumab. This patient experienced resistance to treatment with valacyclovir and prednisone [26]. Adalimumab is a human recombinant immunoglobin G1 monoclonal antibody that binds Tumor Necrosis Factor alpha (TNF-alpha) and neutralizes this membrane soluble receptor so that it can no longer interact with p55 and p57, leading to the induction of apoptosis of TNF-expressing cells. The release of many inflammatory markers like Il-6 and acute phase reactants are induced by TNF-alpha bioactivity [26,27]. It is speculated that TNF-alpha plays a role in drug-induced EM lesions, whereas the lesions from herpes-associated EM is driven predominantly by a delayed hypersensitivity reaction through T-helper 1 cells and interferon-γ. However, this mechanism is not fully elucidated and requires further study [28].

Rituximab has also shown to be beneficial in a case series from 2016 with 5 patients with recurrent severe EM major. All 5 patients had failed therapy with antiviral treatment as well as thalidomide. Four out of 5 patients experienced near complete response to rituximab and another patient experienced a partial response. These four patients that experienced a near complete response had EM major associated with antidesmoplakin autoantibodies, a characteristic that has unknown consequence to the pathogenesis of EM. Rituximab is a chimeric monoclonal antibody that targets the B-cell marker CD20, ultimately leading to apoptosis of these cells and thus suppression of the production of these antibodies; however, the role of B-cell lymphocytes in EM is not yet well understood. These responses to rituximab lasted between 3–11 months. However, disease relapsed in all patients [29].

Another case series from 2017 described the use of apremilast in 3 patients with recurrent oral EM. All 3 patients had failed therapy with antivirals and corticosteroid treatment but experienced complete resolution with no recurrence for up to 6 months following treatment with apremilast [30].

There are no randomized controlled trials to support the efficacy of the treatments described above. Most of these recommendations are derived from case series and expert opinion. Treatment options must be carefully weighed, considering the various adverse effects that are possible with each therapy and their variable efficacies [17].

## 6. Summary

To date, little evidence has been published regarding the treatment of EM. A systematic review from 2019 identified only 1 randomized controlled trial (RCT) and 6 case series that included more than 10 patients [17]. Treatment for acute EM is based on the self-limiting nature of the disease and therefore focuses on symptom control with topical corticosteroids and antihistamines [1,2,3]. For recurrent EM, continuous oral acyclovir has been the only treatment assessed by an RTC. It was shown to suppress recurrence when compared to a placebo [18]. Continuous antiviral therapy is still a first-line therapy for recurrent EM, especially in HSV-induced EM. Additional agents have been trialed in small case studies, with varying degrees of efficacy. Among them, thalidomide, azithromycin, and dapsone, have shown significant rates of complete remission in small case series [1,17]. Immunosuppressants such as adalimumab and rituximab have garnered more interest recently due to their promising success in some case series [27,29]. Lastly, levamisole has shown initial promise in a small cohort of patients [30]. All of these treatment modalities would benefit from assessment in RCTs. High-quality evidence is needed to create a more structured and reliable framework for treating EM.

## Figures and Tables

**Table 1:** A summary of the well-documented trigger factors for Erythema multiforme identified in Sokumbi et al. and Huff et al. [1,4].

Trigger Factors for EM	
Bacterial infections	*Mycoplasma pneumoniae* *Yersinia enterocolitica* *Mycobacterium tuberculosis*
Viral infections	HSV (types 1 and 2) Hepatitis C Virus Epstein–Barr Virus Influenza Virus Cytomegalovirus
Fungal infections	Histoplasma Candida (vulvovaginal candidiasis)
Medications	NSAIDs Antiepileptics Sulfonamides Antibiotics Penicillins
Other conditions	Inflammatory bowel disease Complex apthosis Malignancies Menstruation Benzoic acid consumption Polymorphous light eruption

**Table 2:** A summary of the first-line treatment for each type of EM as well as special considerations that need to be evaluated in each case. Adapted from “Clinical Features, Diagnosis, and Treatment of Erythema Multiforme: A Review for the Practicing Dermatologist” by Sokumbi et al. [1] with permission from the John Wiley and Sons Publishing on behalf of the International Journal of Dermatology, © 2021.

EM Type	First-Line Therapy	Special Considerations
Acute EM	Topical corticosteroids Topical antiseptics	Antiviral therapy in the setting of HSV-associated EM. Antibiotic therapy in the setting of *M. pneumoniae* associated EM.
	Oral antihistamines	Oral methylprednisolone in SARS-CoV-2 associated EM.
Mucosal Involvement	High potency corticosteroid gel	If mucosal involvement is severe, the hospitalization for fluid and electrolyte replacement should be considered in patients with poor oral intake.
	Oral antiseptic or anesthetic solutions	Systemic glucocorticoid therapy, tapered.
		If ocular involvement present, then ophthalmologic evaluation is necessary to prevent serious long-term sequelae.
Recurrent EM	Prophylactic antiviral therapy—topical, continuous oral or intermittent oral (continuous for ≥6 months) acyclovir, valacyclovir, or famciclovir	If resistant to prophylactic antivirals, systemic agents that may be used include: azathioprine, dapsone, mycophenolate mofetil, or immunoglobulin hydroxychloroquine, thalidomide, and cyclosporine.
		For non-responsive EM, another antiviral medication may be substituted, or the dose of the current antiviral doubled.

**Table 3:** This table is an overview of the current recommendations for each type of treatment indicated in the literature [1,20]. Both generic names and brand names, when applicable, are provided. Adapted from “Clinical Features, Diagnosis, and Treatment of Erythema Multiforme: A Review for the Practicing Dermatologist” by Sokumbi et al. [1] with permission from John Wiley and Sons Publishing on behalf of the International Journal of Dermatology, © 2021.

Medication Class	Generic Name	Brand Name
Antibiotic	Azithromycin	Zithromax
	Dapsone	Aczone
Anthelmintic	Levamisole	Ergamisol
Antimalarial	Hydroxychloroquine	Plaquenil
Antihistamine	Cimetidine	N/A
Antiviral	Acyclovir	Acyclovir, Zovirax
	Famciclovir	Famvir
	Valacyclovir	Valaciclovir, Valtrex
Immunosuppressant/immunomodulator	Adalimumab	Humira, Amjevita, and adalimumab-atto
	Apremilast	Otezla
	Azathioprine	Imuran
	Cyclosporine	Ciclosporin, Gengraf, Neoral
	Immunoglobulin	Immune Globulin
	Mycophenolate mofetil	CellCept
	Thalidomide	Thalomid
Steroids	Prednisone	N/A

## Data Availability

Data sharing is not applicable to this article.
